# The need for cultural sensitivity and epistemic justice in applying neuroscience advances in the Global South

**DOI:** 10.3389/fnhum.2026.1715212

**Published:** 2026-02-03

**Authors:** Victor E. Olalde-Mathieu, Giovanna L. Licea-Haquet

**Affiliations:** 1 MIND Research Network, Albuquerque, NM, United States; 2 Universidad Nacional Autónoma de México, Instituto de Neurobiología, Querétaro, México

**Keywords:** cultural sensitivity, epistemic humility, epistemic justice, equity, neuroethics, neurotechnology

This article presents a conceptual and normative argument structured around three core concepts. We define cultural sensitivity as the awareness of cultural biases and the active recognition of different values (Bennett, 1993; Cipta et al., 2024); epistemic justice as the ethical imperative to counter the exclusion of marginalized knowledge in scientific inquiry (Fricker, 2007); and epistemic humility as the recognition of the limits of one's own knowledge (Muyskens et al., 2025). Using these frameworks and drawing on illustrative examples rather than making new empirical claims, the aim of this piece is not to prescribe universal norms, but to encourage dialogue on how these ethical and epistemic commitments can shape more collaborative and contextually grounded scientific practices.

## Neuroscience's current state

Neuroscience is undergoing a technological revolution, with advancements promising to reshape medical practice and human health. Brain-computer interfaces (BCIs), offer new avenues for restoring movement and communication, while algorithmic and AI applications integrated with neuroimaging are beginning to unravel the biological mechanisms of neurodegenerative diseases such as Alzheimer's and Parkinson's. By integrating multimodal data from neuroimaging, electrophysiology, and genomics, these AI-powered systems enable earlier diagnosis, optimize treatment strategies, and personalize interventions, at times approaching specialist-level performance in predicting disease progression (Onciul et al., 2025; Owolabi et al., 2023). These advances hold immense potential to improve outcomes across neurological and psychiatric conditions (Onciul et al., 2025; Owolabi et al., 2023).

At the same time, neuroscience is a rapidly changing field undergoing paradigm shifts (Gong et al., 2024; Knyazev, 2023; Lehmann et al., 2024; Noble et al., 2024; Theriault et al., 2025). The brain's high complexity, together with the need for interdisciplinary, systems-level approaches, has driven the field to reconsider how brain function is studied, encompassing core concepts such as perception, action, cognition, affect, and translation to real world contexts (Barrett et al., 2025; Pessoa et al., 2022; Pezzulo et al., 2024; Yoon et al., 2024). Increasingly, neuroscience recognizes the essential role of variability when studying individuals, mental health and generalizability of frameworks to different cultures (Albantakis et al., 2024; Blevins et al., 2023; Faure et al., 2022; Harnett et al., 2024; Segal et al., 2025). This recognition grounds the possibility that neural and behavioral predictions may not generalize across cultures. It also spotlights the risk that translating frameworks without cultural adaptation could lead to misinterpretation (Chiao, 2018; Chiao et al., 2013; Kitayama and Huff, 2015; Kitayama and Park, 2010).

Given the rapid evolution of neuroscience, it is crucial to recognize the limits of our current knowledge and remain open to revision and alternative perspectives (De Matas et al., 2025; Ramsey, 2021; Rosas et al., 2025). This openness is essential for adapting to new evidence, translating innovations across cultures, and promoting collaborative, interdisciplinary research (Anjum and Aziz, 2024; Porter et al., 2022; Valles, 2018; Vanney et al., 2023).

The current state of neuroscience reveals significant hurdles in translating its advancements to the Global South, particularly concerning equity and governance. As Matshabane et al. (2024) observe, neurotechnologies often disregard sociocultural and infrastructural realities in low-resource settings, leading to uneven access and potential misuse. Similarly, international neurodata initiatives require governance models sensitive to local realities, and equitable technology transfer depends on culturally and institutionally informed policies (Eke et al., 2022; Oyeniji et al., 2025). Compounding these disparities is the risk of “algorithmic colonization”: the uncritical export of data-driven systems built on Global North assumptions into contexts where their embedded biases can reproduce epistemic and social inequalities (Birhane, 2020). In this context, global neuroscience should be guided by cultural sensitivity, epistemic justice and epistemic humility. As Matshabane et al. (2024) and Pant et al. (2022) argue, culturally grounded ethics recognize local values, knowledge systems, and lived experiences as central to equitable implementation. Epistemic justice emphasizes the moral imperative to value diverse ways of knowing and prevent the exclusion of marginalized perspectives from shaping scientific agendas (Dotson, 2012; Fricker, 2007). Epistemic humility, in turn, offers a bridge between ethical awareness and methodological openness, inviting shared governance and plural ways of knowing (Anjum and Aziz, 2024; Porter et al., 2022; Vanney et al., 2023). Taken together, these concepts promote participatory governance that empowers local communities to define the purposes, benefits, and boundaries of technological use (Eke et al., 2022). Without such engagement, technology transfer risks perpetuating new forms of dependency and marginalization (Birhane, 2020).

Given the transdisciplinary nature of the problem, we deliberately use overarching theoretical notions of these concepts. This approach facilitates the integration of diverse knowledge systems needed to address complex socio-technical challenges (Löhr, 2025; Popa et al., 2015). These three concepts, cultural sensitivity, epistemic justice, and epistemic humility, are deeply intertwined, forming a cohesive foundation for ethical innovation. Understanding this framework is crucial for recognizing the perils of its absence.

## Relationally bound pillars of ethical innovation

*Cultural sensitivity*, which involves being aware of the nuances of one's own and other cultures, and the ability to respond to cultural differences in appropriate ways, is crucial for both safety and efficacy (Bennett, 1993; Cipta et al., 2024). For instance, diagnostic tools trained on Western expressions of depression may fail in contexts where distress is expressed somatically (Kirmayer and Ryder, 2016), while therapies based on an individualistic sense of self may be ineffective in collectivist societies (Kitayama and Uskul, 2011). Cultural neuroscience confirms that brain networks related to self-representation and social cognition vary across cultural contexts (Chiao, 2009; Kitayama and Park, 2010). Despite this evidence, global neuroscience remains heavily anchored in Euro-American epistemic frameworks that neglect the sociocultural and linguistic diversity, reducing both the validity and acceptability outside the Global North (Matshabane et al., 2022). Therefore, ethically responsible innovation requires context-sensitive, bidirectional learning and equitable partnerships that integrate local expertise across all stages of design and implementation (Matshabane et al., 2022). Such collaboration not only enhances trust and translational relevance but also fosters equitable access to neuroscientific advances.

*Epistemic justice*, addresses the inclusion of marginalized knowledge systems and the fair distribution of epistemic authority. As Fricker (2007) defines it, epistemic injustice occurs when individuals are wronged in their capacity as knowers, a problem magnified when local or Indigenous perspectives are excluded from defining research priorities. Valuing plural epistemic traditions is essential to enable all communities to participate meaningfully in research and policy (Cummings et al., 2023). Likewise, ethical collaboration in health research requires treating communities as co-producers of knowledge, not as passive data sources (Kaur et al., 2023; Pratt and de Vries, 2023).

These approaches embody epistemic humility, awareness of one's epistemic limits, and openness to learn from others (Muyskens et al., 2025). Such humility exposes how exclusion can persist through procedural norms rather than overt bias. Ferguson (2020) shows that hermeneutical injustice persists when dominant interpretive frameworks obscure marginalized viewpoints, while Schmidt (2024) finds that epistemic exclusion can result from procedural norms that inadvertently silence some participants. Addressing these issues requires intentional practices of inclusion and dialogue, forms of epistemic humility that can reshape how knowledge is produced and shared.

In our view epistemic humility is a cornerstone for ethical innovation in global neuroscience (Gellers, 2024; Hoekstra and Vazire, 2021; Muyskens et al., 2025). It offers a framework for integrating cultural sensitivity and epistemic justice into collaborative practice (Blanco, 2023; Ewuoso, 2023; Walker and Martinez-Vargas, 2022). As a methodological rather than moral stance, humility invites shared governance and plural ways of knowing, fostering a more equitable and context-aware neuroscience, exposing the limits of reductionist assumptions, and reminding us that neuroscience remains interpretive, shaped by cultural logics that guide inquiry and meaning (Knyazev, 2023; Ramsey, 2021; Rosas et al., 2025; Walker and Martinez-Vargas, 2022).

## A scientific imperative for a global neuroscience

Bridging the gap between ethical commitment and scientific practice is essential. From this perspective, epistemic justice is not merely a moral preference but a prerequisite for scientific validity and generalizability (Rebello and Uban, 2023). Thus, ethical reflection and scientific rigor converge. Integrating the Global South into neuroscience is not only an ethical imperative but a scientific one. Historically, the field has relied on data from Western, Educated, Industrialized, Rich, and Democratic (WEIRD) populations (Henrich et al., 2010). Expanding research to diverse groups reveals crucial variations in cognition and brain function, enhancing theoretical accuracy and medical applicability. Neglecting diversity introduces biases that undermine validity. Including underrepresented populations, therefore advances both justice and science (Matshabane et al., 2022; La Scala et al., 2022). In this framework, the Global South is not merely a site for application but a partner in co-creating knowledge and solutions to global neurological and mental health challenges (Matshabane et al., 2024; Oyeniji et al., 2025). This partnership requires epistemic humility to recognize the limits of Western paradigms and learn from other epistemic traditions. Such humility enhances accountability and expands interpretive frameworks, revealing hidden assumptions (Anjum and Aziz, 2024; Hoekstra and Vazire, 2021; Kidd, 2020; Porter et al., 2022). The need is urgent: neurological disorders impose a disproportionate burden on low- and middle-income countries (Feigin et al., 2019; Owolabi et al., 2023), yet, most neuroscientific data still originate from WEIRD samples, undermining the generalizability of findings (Cockcroft, 2025; Henrich et al., 2010).

## The perils of inequitable innovation

Epistemic justice enhances scientific rigor rather than constraining it (Rebello and Uban, 2023). Moreover, without the safeguards of cultural sensitivity and epistemic justice, technological advancement threatens to deepen, rather than alleviate, the global divide. Neglecting cultural sensitivity and epistemic justice in neuroscience creates tangible risks. A primary one is data vulnerability. Neural data, windows into human intentions and emotions, is uniquely sensitive. Without strong, contextually grounded data-sharing and privacy frameworks, this information can be collected, used, and sold without meaningful consent or oversight, exposing individuals to exploitation and populations to profiling (de Lima Dias, 2025; Ienca and Andorno, 2017; Yuste, 2023; Yuste et al., 2017). These risks converge with long-standing concerns about data colonialism and the need for data sovereignty, especially for indigenous and historically marginalized communities (Carroll et al., 2019; Ng, 2025; Walter and Suina, 2019).

A related risk is algorithmic colonization. When technologies trained on WEIRD populations are deployed globally, embedded biases are exported alongside them. Misdiagnoses, skewed predictions, and inequitable access may result, failures already observed in computer vision and clinical algorithms (Atilano-Barbosa and Barrios, 2023; Buolamwini and Gebru, 2018; Gichoya et al., 2022; Henrich et al., 2010; Vyas et al., 2020). Similarly, algorithmic opacity and lack of longitudinal clinical validation can further exacerbate these issues, especially in under-regulated settings (de Lima Dias, 2025; Onciul et al., 2025). For example, commercial vision systems misgender dark-skinned women at thirty-fold higher rates than light-skinned men (Buolamwini and Gebru, 2018), race-corrected equations delay kidney transplantation for Black patients (Vyas et al., 2020) and structural MRI benchmarks derived largely from White samples risk framing typical brain variation in African American populations as abnormal or atypical (Atilano-Barbosa and Barrios, 2023). To address these methodological gaps, recent work like Cardenas-Iniguez and Gonzalez (2024) advocates for a framework for the responsible use of race and ethnicity in neuroimaging that mitigates algorithmic colonization and ensures that models are critically evaluated before being exported across diverse populations. Without participatory governance and rigorous cross-context validation, neuroscience risks becoming a case study in digital neocolonialism, where advances reinforce rather than reduce global inequities (Birhane, 2020; La Scala et al., 2022; Owolabi et al., 2023).

## Concluding reflections

While truly participatory governance, such as community-based participatory research, offers a concrete route to cultural sensitivity and epistemic humility, its implementation is often hampered by limited funding, unequal resource distribution, and institutional inertia that restricts training, mentorship, and sustained engagement with minoritized scholars. Furthermore, there can be potential tensions between universal ethical standards (such as individual data privacy) and local norms that may emphasize collective data stewardship. Acknowledging these difficulties is a core component of epistemic humility; it reinforces that these challenges are not reasons for inaction but are themselves central problems that must be navigated through the very collaborative, contextually grounded dialogue we advocate for throughout this paper.
